# Determinants and disparities in access to paediatricians in Poland

**DOI:** 10.1186/s12875-022-01701-2

**Published:** 2022-04-27

**Authors:** Tadeusz Zienkiewicz, Maria Klatka, Ewa Zienkiewicz, Janusz Klatka

**Affiliations:** 1 Department of Socio-Economic Geography and Spatial Management, Institute of Geography and Environmental Sciences, Jan Kochanowski University of Kielce, Kielce, Poland; 2 Department of Paediatric Endocrinology and Diabetology. Faculty of Paediatrics, Medical University of Lublin, Lublin, Poland; 3 Department of Paediatric Neurology, Faculty of Paediatrics, Medical University of Lublin, Lublin, Poland; 4 Department of Otolaryngology and Laryngeal Oncology, Medical University of Lublin, Lublin, Poland

**Keywords:** Regional diversity, Paediatricians, Healthcare access, Urbanisation, Socioeconomic status, B55, C30, I14, I18, J24, O15

## Abstract

**Background:**

The purpose of this study was to identify the factors that determine the differences in the distribution and workload of paediatricians in Poland. This research, specific to conditions found within Poland, will help further advance knowledge in this area. Data were derived from the database of Statistics Poland. The level of convergence of the phenomenon studied was analysed. The paediatricians' accessibility index was ascertained and its spatial diversity examined. The level of correlation of patients treated per paediatrician was analysed in relation to indices of urbanisation, availability of paediatricians and disposable income.

**Results:**

A moderate variation of patients treated per paediatrician was found and the conditional convergence of the investigated phenomenon observed. A close negative association between the number of patients treated and access to paediatricians (-0.686, *p* = 0.005) was revealed.

**Conclusions:**

The research suggests that socioeconomic factors may affect the uneven spatial distribution of the workload of paediatricians in Poland and cause differences between the provinces in the equal access to paediatricians. This research may thus provide implications for policy and practice as well as lead to a better understanding of the problem.

**Supplementary Information:**

The online version contains supplementary material available at 10.1186/s12875-022-01701-2.

## Introduction

Disparities and inequalities of access to healthcare are the subjects of many studies and analyses. This problem is so complex that social, economic, urban, cultural, racial and demographic factors need to be considered [1–3]. Poland, like most other European countries, is taking action to provide adequate healthcare for its citizens. The challenge is to create a system that guarantees equal access to medical services for each resident. In Poland, difficulties with access to healthcare, especially access to specialist physicians, have been widely recorded [4, 5]. Demographic, economic and social changes taking place in Polish society have resulted in an increased demand for medical services. The pro-family policy implemented by the government, including the "Family 500 + " programme adopted in 2016, is likely to contribute to an increase in the birth rate and thus an increase in demand on the medical services market [6]. The supply of medical services, which is closely related to the number of paediatricians, may become a problem. There were, on average, 80 General paediatricians per 100.000 people under the age of 17 in Poland in 2017 (Greece 227, Irland 38, average in European Union 98) [7]. Their average age was 55.8 years, with a median of 56.0 years. The percentage of paediatricians below 50 years of age was 34.5% [4]. The problem of generational change is very serious because the average annual number of new specialists in paediatrics is only 240 and the retirement age for women is 60 years old and for men 65. The problem of ageing paediatric staff is also affecting other countries [8].

Moreover, the systems and rules for gaining specialisation in Poland are not simple. They change over time and there are many barriers including difficulties in finding places in which to specialise, the privatisation of outpatient healthcare, changes in the form of employment, the lack of employment contracts and the changing rules of contracting by the National Health Fund.

The above examples and other factors, such as the emigration of young doctors [9], have contributed to a reduction in the number of specialised physicians in many areas. As such, it is difficult to envisage a noticeable increase in the number of specialists in Poland. Furthermore, the problem of accessing paediatric healthcare services and the burden placed on paediatricians is gaining importance. The unequal spatial distribution of physicians is not only a problem in Poland. Similar issues have been encountered in all parts of the world [10–13].

The work presented here attempts to determine the factors that affect the spatial inequality and diversity of access to paediatric healthcare in Poland.

## Material and methods

The highest administrative level in Poland, the voivodeship (NUTS-2), was used as the unit of analysis and is hereinafter referred to as “province”.

The database of Statistics Poland (GUS) was the source of statistical data of the number of paediatricians (2010–2017), the number of hospital beds (2010–2017), the population under 17 years of age (2010 and 2017), the index of urbanisation and the disposable income per capita. The number of patients treated per paediatrician (TPP) was calculated by dividing the number of patients 0 – 17 years old treated on paediatric wards (including inter-ward movement) by the population of paediatricians in a given area. The population density of people aged under 17 years of age was calculated as the ratio of the size of this population to the area of the province in which they live [14].

Changes in the spatial distribution of paediatricians from 2010 to 2017 and the disproportionality in paediatric service were analysed. Data on medical personnel working directly with the patient include those working in units in healthcare children’s clubs and social welfare facilities – persons for whom the reporting department is the place of their main employment. Since 2012 this has included departments under the Ministry of National Defence and the Ministry of Interior Affairs. A paediatrician was defined as a physician whose primary specialty is managing medical conditions affecting infants, children and young people.

The study consists of two main stages. In the first stage the variation in the distribution of paediatricians' workload in Poland was determined. The number of patients treated per paediatrician (TPP) was taken as the measure of paediatricians’ workload. For this purpose an analysis of the occurrence of convergence of the studied phenomenon in a given area was carried out.

In the second stage an attempt was made to explain the influence of socio-economic status, level of urbanisation and accessibility to paediatric health care on the level of paediatricians' workload in Poland. For this purpose factors such as Disposable Income per capita (IND), Urbanisation Index (URI) and Paediatric Service Accessibility index (PSA) were extracted. For this purpose, a correlation analysis was performed between selected factors and the level of workload of paediatricians in Poland.

The dynamics of the relative distribution of number of treated patients per paediatrician (TPP) at the level of provinces was analysed, as a first step of the first stage of analysis. The results have been presented in the form of initial and final distribution of relative number of TPP and the change in relative number of TPP by province over the period 2010–2017.

The relative measure of regional differentiation was determined by the coefficient of variation. Sigma-convergence and beta-convergence were assessed. Beta-convergence is defined as the relatively faster development of poorer regions in relation to richer regions, causing a narrowing of the gap between them. Divergence is defined as the opposite phenomenon reflecting the increase in development differences. There are two main concepts of convergence in the literature: sigma and beta-convergence [15, 16]. The first one, sigma-convergence, relates to changes in income distribution (or other phenomenon) over time. It occurs when the dispersion of per capita income (or other phenomenon) between regions decreases over time. The concept of beta-convergence refers to the relationship between the average growth rate of tested phenomenon and its initial level [15]. In the literature it appears in two variants. Absolute convergence assumes that poorer regions develop faster than richer ones, and that the lower the starting level, the greater their growth in real GDP per capita. That means the regions become similar to each other regardless of the initial conditions. Conditional convergence, on the other hand, means that regions with similar structural parameters become more similar to each other.

Thus, regions with different characteristics converge to different income levels. Beta-convergence is related to income mobility between regions within the same distribution. Beta-convergence is necessary but not sufficient for sigma-convergence to occur.

To explain the disproportion in access to paediatric healthcare services, the following indicators were used: Disposable Income (IND), the Urbanisation Index (URI), the Paediatric Service Accessibility index (PSA) and the number of patients treated per 100,000 inhabitants under 17 years of age on public paediatric wards (TPP) for 2017.

The Urbanisation Index (URI) and the number of patients treated per 100,000 inhabitants under 17 years of age on public paediatric wards (TPP) for 2017 were obtained from the Central Statistical Office database.

Access to paediatricians, in this study, was defined by the Paediatric Service Accessibility index. It is known that the access of a specific consumers' target group to any goods or services is influenced by several heterogeneous factors. These may include the communication infrastructure and its resources, the infrastructure directly related to the good or service offered, the consumer's purchasing power, the size of possible subsidies or support from local authorities, as well as the size of the consumer group. The construction of the PSA measure was a multi-step process. The first step in this regard was the selection of output variables. The authors made every effort to ensure that variables were measurable, logically connected with the complex phenomenon under consideration and characterised by such properties as: adequate information quality and diversity, relevance from the point of view of the analysed phenomena, completeness of information on all aspects significantly influencing the shape of the given complex phenomenon, unambiguity and precision of definition or interrelation from the logical point of view. The Paediatric Service Accessibility index (PSA) was calculated using statistical variables supplied by the Central Statistical Office of Poland—Local Data Bank database (Table 1).

**Table 1:** Variables for 2017

Symbol	Name	Measure	Nature of the variable
*X1*	The population aged 0–17	person	Stimulant
*X2*	the paediatrician workforce	persons per 100.000 residents under 17 years of age,	Stimulant
*X3*	patients treated on paediatric wards (including inter-ward movement)	person	Stimulant
*X4*	the average disposable income per capita	PLN	Stimulant
*X5*	outpatient departments	number	Stimulant
*X6*	paramedic teams	number	Stimulant
*X7*	the average monthly gross wages and salaries in human health and social work activities	PLN	Stimulant
*X8*	railway lines operated	number per 100 km^2^	Stimulant
*X9*	hard surface public roads	number of total per 100 km^2^	Stimulant
*X10*	passenger cars	number per 1.000 population	Stimulant
*X11*	national regular transport lines	km	Stimulant
*X12*	expenditure of provinces relating to healthcare	in mln PLN	Stimulant

*Note:* author’s work based on GUS Local Data Bank database

The selection of diagnostic variables for the determination of the Paediatric Service Accessibility index was made taking into account: a) economic factors (average disposable income per capita, average gross monthly remuneration in activities related to human health and social work, provincial expenditures related to health care); b) infrastructural factors enabling patients to reach paediatricians (operated railway lines, hard-surfaced public roads, passenger cars, national regular transport lines); c) medical resources (paediatric staff, patients treated in paediatric wards (including interdepartmental traffic), outpatient departments, emergency medical teams).

The second stage of analysis consisted in verification of variables. Its aim was to extract from among collected variables those which, from the point of view of the considered complex phenomenon, bring the greatest informative-differentiating value in relation to knowledge about considered objects. The verification was carried out in two steps. First, selection was made in terms of variability. Variables with too low variability (diversity) were eliminated, i.e. those which show too low differentiating power among examined objects. Variables, for which absolute value of coefficient of variation was below 0.1 (10%) were eliminated.

The variables left in the model were subjected to correlation verification. The purpose of such action is to eliminate data that are excessively correlated, i.e. they carry similar information as others. Therefore, correlation coefficients of all pairs of variables should be taken into account. The starting point here is the determination of the correlation matrix of the variables.

In order to achieve integrity of the taxonomic model, inverse correlation matrix method was applied [17, 18]. It consists in determining the inverse matrix to Pearson's correlation matrix of variables R, i.e. R^−1^. Then, the diagonal elements of this inverse correlation matrix R^−1^ are examined. If the variables do not show many close correlations, its diagonal elements are Variance Inflation Factor (VIF) for the given variables in comparison with others and are:

1 $${VIF}_{j}=\frac{1}{1-{R}_{j}^{2}}$$

where *R*_*j*_—regression coefficient of determination of the *j*-th variable against the others, *j* = 1, 2, …, p, *p*—the number of variables in the model after the volatility verification[18]. From formula (1) it follows that diagonal elements of R^−1^ matrix should should belong to the interval [1, ∞). If these elements are too large, e.g. greater than 10—it means faulty numerical conditioning of the R^−1^ matrix, that is excessive correlation of a given variable with the others. As a result of variation and correlation verification, a set of diagnostic variables X2, X3, X4, X5, X8, X9, X11, X12 was formed.

The proper construction of a taxonomic measure required the stimulation and normalization of selected variables. For this purpose, the direction of influence of variables on the status of objects from the point of view of the considered complex phenomenon was identified. By direction, diagnostic variables are divided into: a) stimulants—variables whose higher value indicates a better position of the object in a given context; b) destimulants—the higher the value of a given variable, the worse the position of the object in the phenomenon under consideration; c) nominants—variables having an optimal level of value (inflection point), below which they are stimulants and above which they are destimulants, or vice versa.

The selected variables turned out to be stimulants.

Normalization is reduction (usually expressed in different units of measure and having different range of values) of diagnostic variables to the best, comparable form, e.g. through standardization, unitarization or quotient transformations [17, 18]. In this study, standardization of variables was used.

A numerical description of the set of objects was made using the observation matrix:

2 $$X=\left[\begin{array}{ccc}{x}_{11}& \cdots & {x}_{1m}\\ \vdots & \ddots & \vdots \\ {x}_{n1}& \cdots & {x}_{nm}\end{array}\right],$$

where: *x*_*ij*_ – the value of *j*-th characteristic for *i*-th object ( *i* = 1, 2,…, *n*; *j* = 1, 2,…, *m*).

Because of the variables chosen, the normalisation of selected variables was made using the method of standardisation with the following formula:

3 $${z}_{ij}=\frac{{x}_{ij}-{\overline{x} }_{j}}{{S}_{xj}},$$

As a result of these transformations a matrix of standardised values of characteristic Z was created:

4 $$Z=\left[\begin{array}{ccc}{z}_{11}& \cdots & {z}_{1m}\\ \vdots & \ddots & \vdots \\ {z}_{n1}& \cdots & {z}_{nm}\end{array}\right],$$

where *z*_*ij*_ – the standardised values of *x*_*ij*_*.*

The Paediatric Service Accessibility index was used as the indicator of access to paediatric healthcare and was calculated using the Hellwig’s method [17, 18]. After determining the pattern of access to medical services, ${z}_{0j}=\underset{i}{\mathrm{max}}{z}_{ij}$ where “*j”* is a stimulant, the taxonomic distances between the individual and the model object were established. The average values of each feature were used to determine the model. The maximum PSA index for the model object is equal 1. The synthetic measure for each unit is described by Hellwig’s synthetic indicator (*d*_*i*_) and was determined using the following formula:

5 $${d}_{i}=1-\frac{{c}_{i0}}{{c}_{0}}$$

where, *d*_*i*_—a measure of accessibility; *c*_*i0*_—taxonomic distance of each unit *z*_*ij*_ to model object *z*_*0j*_; *c*_*0*_—a critical distance from the model of the unit. Used multiplicities are expressed as:

6 $${c}_{i0}=\sqrt{\sum_{j=1}^{n}{\left({z}_{ij}-{z}_{oj}\right)}^{2}}$$

where

7 $${c}_{0}=\overline{{c }_{0}}+2{S}_{d}$$

and

8 $$\overline{{c }_{0}}=\frac{1}{n}\sum_{j=1}^{n}{c}_{i0}$$

and

9 $${S}_{d}=\sqrt{\frac{1}{n}\sum_{j=1}^{n}{\left({c}_{i0}- {c}_{0}\right)}^{2}}$$

The transformed indicator for access to paediatric healthcare is assigned a value of 100. Hellwig’s synthetic indicator (d_i_) was calculated for each object (province) and a positive value from 0 to 1 was assumed. The higher the level of synthetic measure the more favourable the situation of the object. Next, the objects were ordered linearly, classified in four typological classes and collected in separate groups of similar objects. The final classification was determined on the basis of the mean value of the synthetic indicator ($\overline{{{\varvec{d}} }_{{\varvec{i}}}}$) and its standard deviation (*S*_*di*_).

Group I: the synthetic indicator value: ${{\varvec{d}}}_{{\varvec{i}}}>\overline{{{\varvec{d}} }_{{\varvec{i}}}}+{{\varvec{S}}}_{{\varvec{d}}{\varvec{i}}};$

Group II: the synthetic indicator value: $\overline{{{\varvec{d}} }_{{\varvec{i}}}}\boldsymbol{ }<{{\varvec{d}}}_{{\varvec{i}}}\boldsymbol{ }\le \boldsymbol{ }\overline{{{\varvec{d}} }_{{\varvec{i}}}}+{{\varvec{S}}}_{{\varvec{d}}{\varvec{i}}};$

Group III: the synthetic indicator value: $\overline{{{\varvec{d}} }_{{\varvec{i}}}}-{{\varvec{S}}}_{{\varvec{d}}{\varvec{i}}}<{{\varvec{d}}}_{{\varvec{i}}}\le \overline{{{\varvec{d}} }_{{\varvec{i}}}}$

Group IV: the synthetic indicator value: ${{\varvec{d}}}_{{\varvec{i}}}\le \overline{{{\varvec{d}} }_{{\varvec{i}}}}-{{\varvec{S}}}_{{\varvec{d}}{\varvec{i}}}$

## Results

**Fig. 1:**
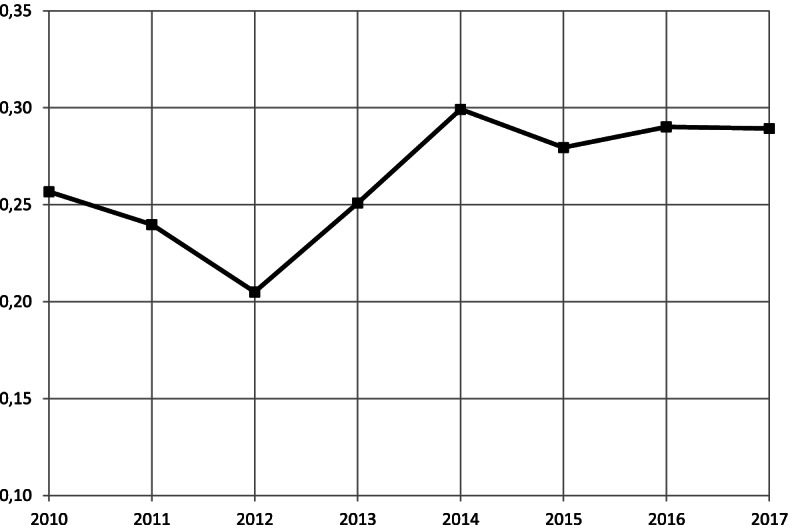
The curve of measure of relative regional differences for patients treated per paediatrician in the period of 2010 –2017 on paediatric wards (including inter-ward movement)**.** Note: own elaboration

In order to determine the existence and scale of dispersion of the number of patients treated per one paediatrician, a coefficient of variation as a measure of relative regional differences of the above variable was determined and is expressed in Fig. 1.

The results show that the relative regional differences in the study area among patients treated per paediatrician in the period of 2010 -2017 on paediatric wards, remains at an average level (25%—45%). Low variation of the coefficient of variation of the examined variable was recorded in the period 2011 -2013. The results suggest the presence of moderate variation in the distribution of the workload of paediatricians in Poland.

**Fig. 2:**
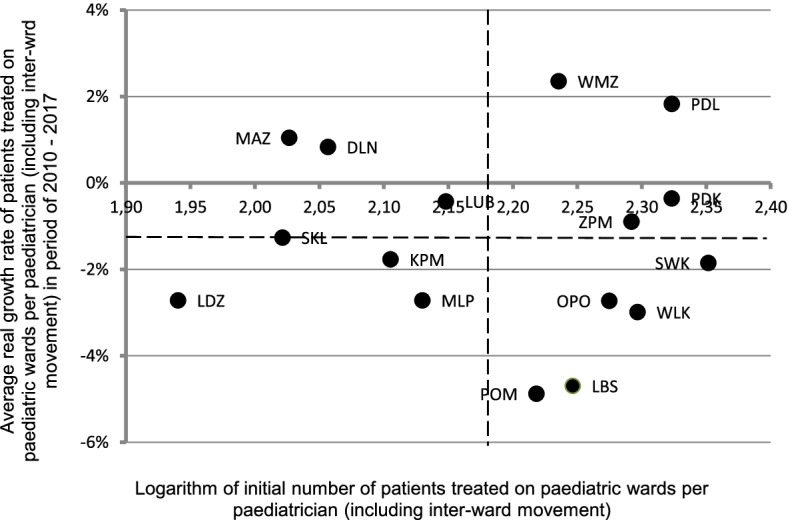
The conditional beta-convergence of patients treated per paediatrician.Legend: The horizontal dashed line indicates the mean value for average real growth rate of patients treated on paediatric wards per paediatrician, (-1.3%) and the dashed horizontal line indicates the mean value for logarithm of initial number of patients treated per paediatrician on paediatric wards (2.18). DLN: Dolnośląskie; KPM: Kujawsko-Pomorskie; LUB: Lubelskie; LBS: Lubuskie; LDZ: Łódzkie; MLP: Małopolskie; MAZ: Mazowieckie; OPO: Opolskie; PDK: Podkarpackie; PDL: Podlaskie; POM: Pomorskie; WMZ: Warmińsko-Mazurskie; SKL: Śląskie; SWK: Świętokrzyskie; WKL: Wielkopolskie; ZPM: Zachodniopomorskie. Note: own elaboration based on [12]

Therefore, an analysis of convergence of the number of patients treated by one paediatrician was performed (Fig. 2).

The performed analysis showed that the average real growth rate in the number of patients treated in paediatric wards per paediatrician (including interdepartmental movement) decreased between 2010 and 2017 o 1,3%.

Some provinces with a high initial logarithm of patients treated on paediatric wards per paediatrician experienced an increase in the average real rate of patients treated per paediatrician whilst some from the same group of provinces noted a decrease in the above rate.

A similar situation was observed in a group of provinces with a lower than average initial logarithm of patients treated on paediatric wards per paediatrician. In this group, there were provinces which saw a real increase in the rate of patients treated by one paediatrician as well as those where a decrease in the rate was observed.

Given the above, it should be noted, that in the period between 2010 – 2017, the conditional convergence in terms of the number of patients treated per paediatrician was observed in Poland (Fig. 2).

The possibility of providing any services, including medical services, is also associated with the possibility of access to the mentioned service. In this paper, the ability of a population under 17 years old to access public paediatric services is considered by setting out an appropriate Paediatric Service Accessibility index (PSA).

Table 2 presents values of Disposable Income per capita (IND), Urbanisation Index (URI), Paediatric Service Accessibility index (PSA) and the number of inhabitants under 17 years of age treated per paediatrician on public paediatric wards (TPP) in 2017. Disparities in access to public paediatric healthcare according to PSA can be observed.

**Table 2:** The values of variables describing the regional diversity

**Province**	Disposable Income per capita in PLN	Urbanisation Index	Paediatric Service Accessibility index	The number of treated patients per paediatrician
Dolnośląskie	1 626	0.688	0.68	118
Kujawsko-Pomorskie	1 457	0.593	0.63	109
Lubelskie	1 437	0.465	0.56	135
Lubuskie	1 591	0.649	0.57	125
Łódzkie	1 566	0.627	0.64	70
Małopolskie	1 492	0.483	0.60	110
Mazowieckie	1 912	0.643	0.77	112
Opolskie	1 511	0.528	0.57	150
Podkarpackie	1 254	0.412	0.59	158
Podlaskie	1 586	0.607	0.54	228
Pomorskie	1 649	0.639	0.64	113
Śląskie	1 646	0.769	0.70	96
Świętokrzyskie	1 433	0.446	0.58	194
Warmińsko-Mazurskie	1 496	0.590	0.56	187
Wielkopolskie	1 607	0.546	0.65	157
Zachodniopomorskie	1 653	0.686	0.59	183

*Note:* own elaboration based on [14]

The highest level of paediatric service accessibility was achieved by Group I provinces of Mazowieckie and Śląskie. Group II contained five provinces (Dolnośląskie, Wielkopolskie, Łódzkie, Pomorskie, Kujawsko-Pomorskie) which had a relatively high level of PSA. Group III included six provinces (Małopolskie, Zachodniopomorskie, Podkarpackie, Świętokrzyskie, Opolskie, Lubuskie) which were characterised by low levels of the chosen indicator. The lowest level of paediatric service accessibility was presented by three provinces (Warmińsko-Mazurskie, Lubelskie, Podlaskie) which made up Group IV (Table 3).

**Table 3:** The classification of provinces by the Paediatric Service Accessibility index

Group	Ranges	Provinces
I	di > 0.68	Mazowieckie, Śląskie
II	0.62 < di < = 0.68	Dolnośląskie, Wielkopolskie, Łódzkie, Pomorskie, Kujawsko-Pomorskie,
III	0.56 < di < = 0.62	Małopolskie, Zachodniopomorskie, Podkarpackie, Świętokrzyskie, Opolskie, Lubuskie
IV	di < = 0.56	Warmińsko-Mazurskie, Lubelskie, Podlaskie

Note: own elaboration

Table 4 presents the correlation between the noted variables and suggests that there is a statistically significant (0.724, *p* < 0.002) linear relationship between disposable income and the Urbanisation Index (URI). The accessibility to paediatric healthcare services, represented by PSA, also shows a statistically significant correlation with disposable income (0.686, *p* = 0,004). No statistically significant relationship was discovered between accessibility to paediatric healthcare services and the urbanisation rate of provinces. The level of paediatric workload, represented by TPP, negatively correlates with the availability of public paediatric services accessibility (–0.618, *p* = 0.005). This correlation is statistically significant. There was no statistically significant correlation between the level of paediatric occupancy, disposable income and the level of urbanisation, and neither was there between the availability of paediatric services and the level of urbanisation.

**Table 4:** Table of correlation

Variable	IND	URI	PSA	TPP
IND	1.000	0.724*	0.686*	-0.239
	*p* = –-	*p* = 0.002	*p *= 0.004	p = 0.178
URI	0.724*	1.000	0.523	-0.313
	*p* = 0.002	*p* = –-	p = 0.092	*p *= 0.234
PSA	0.686*	0.523	1.000	-0.618*
	*p* = 0.004	*p* = 0.092	*p* = –-	*p* = 0.005
TPP	0.239	-0.313	-0,618*	1.000
	*p* = 0.178	*p* = 0.234	*p* = 0.005	*p* = –-

^*^—Statistical significance. Note: own elaboration

Correlation analysis revealed a high, statistically significant positive correlation between per capita income and level of urbanization. Income also correlates strongly and statistically significantly with the rate of access to paediatric health care services. This situation suggests that patients with high economic status have easier access to pediatric services. The presence of a strong and statistically significant negative correlation between the TPP index and the paediatric service availability index suggests a higher workload for paediatricians in areas with low paediatric service availability.

## Discussion

During the research carried out into the incidence of variation in access to paediatric services, it was explained that Poland is not a homogenous country in this respect. There is moderate variation across the country in the number of patients served per paediatrician. This situation has a direct impact on the different levels of paediatricians' workload. It was observed that not all provinces with a high logarithm of initial number of patients treated on paediatric wards per paediatrician recorded a growth rate of patients treated on paediatric wards per paediatrician in the period of 2010—2017. In some of these provinces, the workload of paediatricians fells below the average during the period studied. On the other hand, provinces with a low initial logarithm of TPP recorded an increase in the number of patients served per paediatrician. In this case, the provinces of Mazowieckie and Dolnośląskie are worth mentioning.

**Fig. 3:**
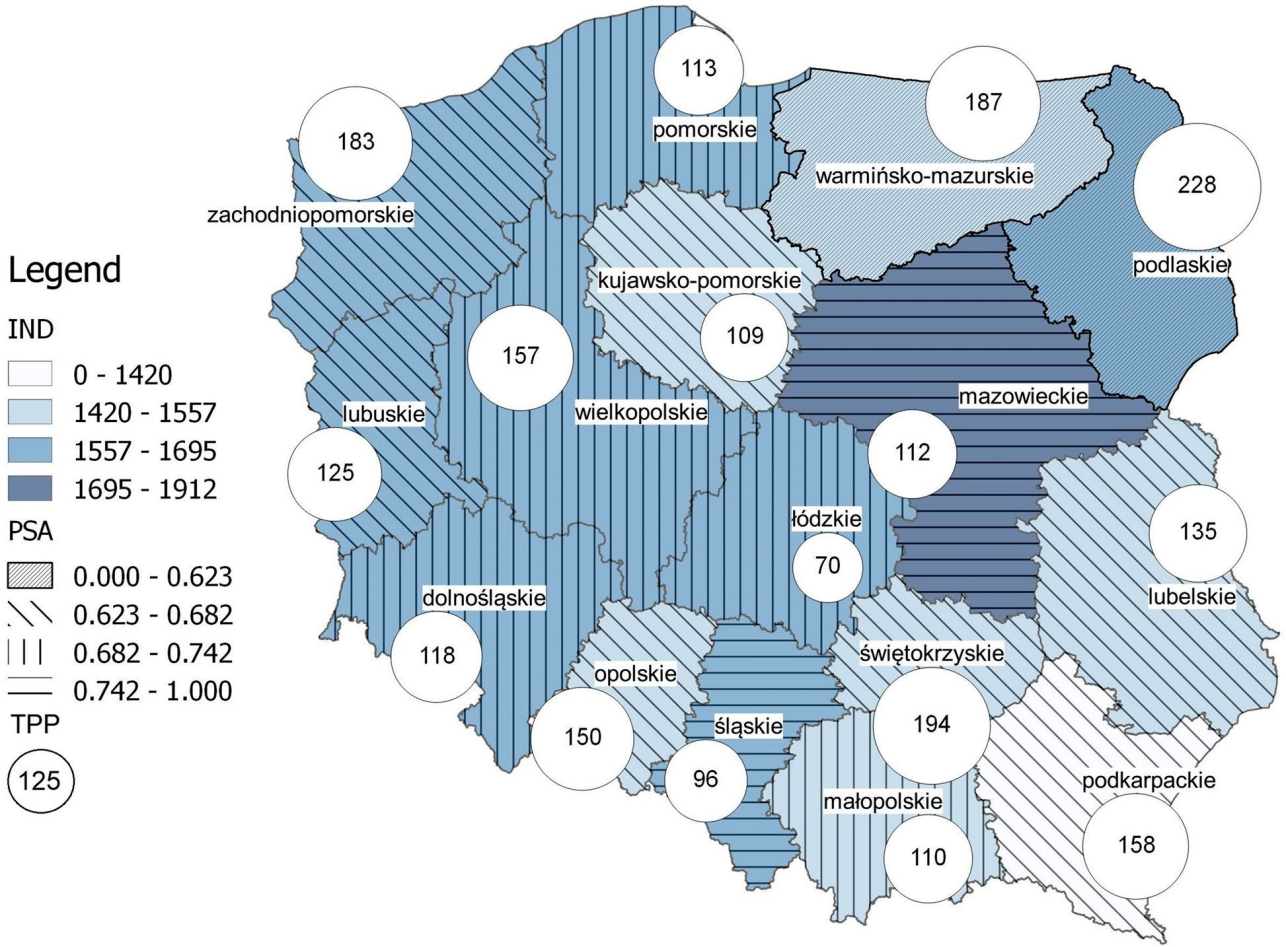
Workload of paediatricians in relation to a background of socioeconomic status and access to paediatric healthcare in Poland in 2017.Legend: IND – Disposable income per capita in PLN, PSA – Paediatric Service Accessibility index, TPP – Number of treated patients per paediatrician. Note: own elaboration using Quantum GIS 2.8 Wien

However, no region with a high level of access to paediatric services and a high socioeconomic status can be identified in Poland. Zachodniopomorskie province is characterised by the one of the high level of disposable income, but, at the same time, has the one of the lowest level of access to paediatric services. Which, in light of the strong and positive correlation between IND and PSA, may seem incomprehensible. This situation may be the result of the economic migration of Polish doctors to Germany [5]. A similar situation might account for what is happening in Lubuskie and Dolnośląskie provinces (Fig. 3). We observed that seven out of eleven border provinces present the lowest level of access to paediatric services. In some of them: Podlaskie, Warmińsko-Mazurskie, Zachodniopomorskie and Podkarpackie, the highest paediatrician workload rates are recorded. The high level of this indicator is also found in the Świętokrzyskie province (194). Nevertheless, a very wide variation in paediatricians’ workload is observed which can reach up to 300%. The smallest number of paediatric patients treated by one paediatrician was recorded in Łódzkie (70) and the highest in Podlaskie (228). The higher number of patients treated per paediatrician suggests the lack of specialists, which has a significant impact on access to paediatric care. This can be clearly seen in regions with a high level of disposable income (Zachodniopomorskie), where the number of patients treated per paediatrician is one of the highest in Poland, versus those provinces which have a low level of disposable income (Świętokrzyskie, Podkarpackie, Lubelskie). The reasons for this phenomenon could be the migration of physicians [7, 19]. The demand on the labour market as well as financial and non-financial benefits (fast-track to specialisation, free accommodation) might also affect their decision about choosing a workplace. As early as during their internship, physicians mostly choose to work in the main cities of regions where medical and other universities can be found, or they even try to start their career abroad  [7]. The economic migration of physicians from Poland is not an isolated phenomenon and concerns many countries, especially developing ones [20, 21].

People living in under-developed regions are usually more socially disadvantaged and so tend to require greater access to healthcare. They use more acute hospital care and less primary care or private medical service than patients with a higher socioeconomic status [3–23].

Patients look for factors described by Thomas and Penchansky such as accessibility, accommodation, acceptability and competence in clinical care [24, 25]. Hospital care in Poland is perceived as more specialised and cheaper than outpatient care. Outpatient care does not guarantee such a wide spectrum of free diagnostic tests as provided by a hospital. This results in additional time and expense for check-ups recommended by the general practitioner (GP). The population under the age of 18 in Poland benefits from being able to access the healthcare system free of charge. However, a GP is only allowed to prescribe a closed catalogue of necessary check-ups free of charge. More advanced follow-ups and diagnostic tests (e.g. magnetic resonance imaging, computed tomography) are payable by the patient. In addition to the above, outpatient care is commonly recognised as less accessible than hospital care, especially in underdeveloped areas where road network density is low and public transport unreliable.

Most of the citizens living in under-developed provinces, with a low level of income, cannot afford regular outpatient appointments because of the time involved and, in particular, the fees for diagnostic tests. Patients do not want to waste time searching for a specialist. They expect the specialist to care for them. A stay in a hospital guarantees such a service. Considering accessibility, hospital-based care is more convenient than an outpatient one. Parents of small children usually work during regular office hours which makes it challenging to access outpatient services during that time. The hospital relieves them of this problem. And besides, they are allowed to claim payable sick leave for the duration of the child's stay in hospital.

Finally, the adoption of the new "Act on Publicly Funded Health and Personal Healthcare Services" and the reform of the Night and Holiday Medical Care, has led to contact points being located in hospitals, often in emergency departments [26]. This has contributed to an increased number of hospitalised patients. The previous system emphasised substitution treatment by the general practitioner, who only in very demanding cases referred the patient to hospital. The new system creates a situation where the patient is, for all intents and purposes, already in hospital. In addition to the above, there are many insurance companies active in economically developed areas, guaranteeing round-the-clock medical care for a small monthly fee. In less developed regions, not attractive for medical business, the population is very poorly served by private medical services including paediatric ones.

Populations living in under-developed regions, with lower disposable income per capita, mostly use public healthcare because of a lack of alternative means. Physicians in economically well-developed regions spend more of their working time in private clinics or outpatient care facilities than in public healthcare units, even though the latter are usually their primary place of work. The combination of economic, social and organisational factors creates a specific social norm regarding the acceptability of hospital versus outpatient care. A hospital is viewed as the place to go to when one is sick but primarily because medical check-ups are provided free of charge.

Taxonomic analysis of access to paediatric medical services has shown that areas with a high level of disposable income per capita are characterised by better access to these kinds of paediatric services. This situation is also related to the fact that there is a network of medical schools, including universities, in most principal cities of provinces with a high level of socioeconomic development. The high supply of medical services contributes to a more efficient use of medical staff in these provinces than in areas without medical education establishments. The existence of better medical care in regions with a high level of socioeconomic development is an indisputable fact [27].

The research presented in this paper, referring specifically to Poland, builds upon our basic understanding of the subject [5, 28] and helps to fill a gap in the existing literature.

## Conclusion

This paper focuses on the equity of access to paediatric healthcare resources as one of the main factors of sustainable development and a healthy society. The regional disparities in the allocation and unequal workload of paediatricians may create a significant obstacle or even completely exclude part of the younger population from accessing the healthcare system. The results suggest that a low level of socioeconomic development may be one of the factors causing high demand for public paediatric healthcare, thereby increasing the workload of paediatricians and posing a threat to the quality of healthcare services in underdeveloped provinces. The public paediatric workforce with the highest workload is seen in economically underdeveloped provinces with the lowest disposable income. Well-developed provinces attract paediatricians and the population benefits from easier access to paediatric healthcare. The elimination of inequalities, including geographical imbalances, in access to the healthcare system is an important element in a country’s healthcare policy. The research presented in this paper can contribute to the formulation of a healthcare policy to negate the factors that cause an unequal distribution of paediatricians and to ensure equal access to healthcare for children and adolescents.

Conducting additional research, using other methods e.g. Principal Component Analysis and covering other types of factors, including social, cultural and infrastructural, would broaden our knowledge on their impact on patient accessibility to a given health care sector in Poland.

## Supplementary Information

**Additional file 1.** 1 Number of paediatricians and patients treated on paediatric wards per paediatrician 2010 – 2017

**Additional file 2.** A – 2 Number of patients treated on paediatric wards per paediatrician 2010 – 2017 and ß-convergence characteristics

**Additional file 3.** A – 3 Attributive variables (2017)

**Additional file 4.** A – 4A The taxonomic accessibility model variables

## Data Availability

All data generated or analysed during this study are included in this published article. The data may be found: http://swaid.stat.gov.pl/SitePages/StronaGlownaDBW.aspx

## Abbreviations

GDP: Gross Domestic Product
GP: General Practitioner
GUS: Statistics Poland
IND: Disposable Income
SES: Socioeconomic Status
PLN: Polish currency (zloty)
PSA: Paediatric Service Accessibility index
TPP: Number of treated patients per paediatrician
UNDP: United Nations Development Programme
URI: Urbanisation Index
