# Supplementary material for: Determinants and disparities in access to paediatricians in Poland
Source: BMC Prim Care. 2022 Apr 27;23:94. doi: 10.1186/s12875-022-01701-2 (PMC9044810; doi:10.1186/s12875-022-01701-2)
Supplement: Supplementary file 1 — Additional file 1. 1 Number of paediatricians and patients treated on paediatric wards per paediatrician 2010 – 2017 [file 12875_2022_1701_MOESM1_ESM.docx]

A – 1 Number of paediatricians and patients treated on paediatric wards per paediatrician 2010 – 2017

| **Voivodship** | **Number of paediatricians** | | | | | | | | **Treated on paediatric wards per paediatrician**  **(including inter-ward movement)** | | | | | | | |
| --- | --- | --- | --- | --- | --- | --- | --- | --- | --- | --- | --- | --- | --- | --- | --- | --- |
|  | 2010 | 2011 | 2012 | 2013 | 2014 | 2015 | 2016 | 2017 | 2010 | 2011 | 2012 | 2013 | 2014 | 2015 | 2016 | 2017 |
| Dolnośląskie | 263 | 274 | 253 | 271 | 275 | 265 | 286 | 265 | 114 | 108 | 115 | 115 | 113 | 116 | 102 | 118 |
| Kujawsko-pomorskie | 210 | 194 | 218 | 235 | 209 | 232 | 242 | 235 | 127 | 140 | 142 | 139 | 151 | 129 | 113 | 109 |
| Lubelskie | 159 | 149 | 135 | 143 | 141 | 146 | 148 | 143 | 141 | 158 | 161 | 160 | 160 | 158 | 147 | 135 |
| Lubuskie | 63 | 69 | 70 | 77 | 78 | 80 | 82 | 86 | 176 | 157 | 150 | 148 | 150 | 152 | 144 | 125 |
| Łódzkie | 313 | 296 | 283 | 282 | 291 | 283 | 325 | 335 | 87 | 92 | 92 | 96 | 90 | 85 | 76 | 70 |
| Małopolskie | 255 | 273 | 259 | 274 | 279 | 286 | 322 | 331 | 135 | 127 | 130 | 128 | 123 | 123 | 107 | 110 |
| Mazowieckie | 464 | 465 | 504 | 517 | 544 | 533 | 562 | 573 | 106 | 122 | 121 | 121 | 115 | 113 | 105 | 112 |
| Opolskie | 70 | 70 | 71 | 66 | 69 | 78 | 88 | 76 | 188 | 193 | 170 | 195 | 182 | 164 | 140 | 150 |
| Podkarpackie | 172 | 162 | 174 | 176 | 186 | 204 | 207 | 188 | 167 | 181 | 165 | 170 | 162 | 142 | 136 | 158 |
| Podlaskie | 103 | 104 | 111 | 97 | 82 | 88 | 95 | 87 | 210 | 206 | 189 | 228 | 269 | 239 | 218 | 228 |
| Pomorskie | 200 | 196 | 188 | 180 | 210 | 239 | 261 | 244 | 165 | 161 | 155 | 167 | 140 | 121 | 106 | 113 |
| Śląskie | 390 | 393 | 373 | 369 | 378 | 383 | 398 | 404 | 105 | 102 | 103 | 108 | 106 | 108 | 104 | 96 |
| Świętokrzyskie | 96 | 98 | 100 | 93 | 96 | 94 | 102 | 104 | 225 | 224 | 205 | 231 | 226 | 224 | 208 | 194 |
| Warmińsko-mazurskie | 119 | 124 | 114 | 103 | 102 | 110 | 136 | 122 | 172 | 166 | 177 | 197 | 229 | 188 | 153 | 187 |
| Wielkopolskie | 203 | 212 | 237 | 218 | 252 | 223 | 232 | 243 | 198 | 189 | 168 | 182 | 166 | 175 | 168 | 157 |
| Zachodniopomorskie | 142 | 145 | 138 | 135 | 129 | 134 | 138 | 148 | 196 | 187 | 182 | 195 | 202 | 205 | 198 | 183 |
| Statistic parameter | | | | | | | | | | | | | | | | |
| Mean | 210 | 202 | 202 | 202 | 208 | 211 | 227 | 224 | 157 | 157 | 152 | 161 | 161 | 153 | 139 | 140 |
| Standard deviation | 108 | 110 | 113 | 117 | 124 | 121 | 128 | 132 | 40 | 38 | 31 | 40 | 48 | 43 | 40 | 41 |
| Coefficient of variation | 0,51 | 0,55 | 0,56 | 0,58 | 0,60 | 0,57 | 0,56 | 0,59 | 0,26 | 0,24 | 0,20 | 0,25 | 0,30 | 0,28 | 0,29 | 0,29 |
| Minimum | 63 | 69 | 70 | 66 | 69 | 78 | 82 | 76 | 87 | 92 | 92 | 96 | 90 | 85 | 76 | 70 |
| Maximum | 464 | 465 | 504 | 517 | 544 | 533 | 562 | 573 | 225 | 224 | 205 | 231 | 269 | 239 | 218 | 228 |
