# Supplementary material for: Determinants and disparities in access to paediatricians in Poland
Source: BMC Prim Care. 2022 Apr 27;23:94. doi: 10.1186/s12875-022-01701-2 (PMC9044810; doi:10.1186/s12875-022-01701-2)
Supplement: Supplementary file 2 — Additional file 2. A – 2 Number of patients treated on paediatric wards per paediatrician 2010 – 2017 and ß-convergence characteristics [file 12875_2022_1701_MOESM2_ESM.docx]

A – 2 Number of patients treated on paediatric wards per paediatrician 2010 – 2017 and ß-convergence characteristics

| **Voivodship** | **Treated on paediatric wards per paediatrician** | | | | | | | | Logarithm of initial number of patients treated on paediatric wards per paediatrician | Average real growth rate of patients treated on paediatric wards per paediatrician |
| --- | --- | --- | --- | --- | --- | --- | --- | --- | --- | --- |
|  | **(including inter-ward movement)** | | | | | | | |  |  |
|  | 2010 | 2011 | 2012 | 2013 | 2014 | 2015 | 2016 | 2017 |  |  |
| Dolnośląskie | 114 | 108 | 115 | 115 | 113 | 116 | 102 | 118 | 2,057 | 0,008 |
| Kujawsko-pomorskie | 127 | 140 | 142 | 139 | 151 | 129 | 113 | 109 | 2,104 | -0,018 |
| Lubelskie | 141 | 158 | 161 | 160 | 160 | 158 | 147 | 135 | 2,149 | -0,004 |
| Lubuskie | 176 | 157 | 150 | 148 | 150 | 152 | 144 | 125 | 2,246 | -0,046 |
| Łódzkie | 87 | 92 | 92 | 96 | 90 | 85 | 76 | 70 | 1,940 | -0,029 |
| Małopolskie | 135 | 127 | 130 | 128 | 123 | 123 | 107 | 110 | 2,130 | -0,027 |
| Mazowieckie | 106 | 122 | 121 | 121 | 115 | 113 | 105 | 112 | 2,025 | 0,010 |
| Opolskie | 188 | 193 | 170 | 195 | 182 | 164 | 140 | 150 | 2,274 | -0,027 |
| Podkarpackie | 167 | 181 | 165 | 170 | 162 | 142 | 136 | 158 | 2,223 | -0,004 |
| Podlaskie | 210 | 206 | 189 | 228 | 269 | 239 | 218 | 228 | 2,322 | 0,019 |
| Pomorskie | 165 | 161 | 155 | 167 | 140 | 121 | 106 | 113 | 2,217 | -0,048 |
| Śląskie | 105 | 102 | 103 | 108 | 106 | 108 | 104 | 96 | 2,021 | -0,012 |
| Świętokrzyskie | 225 | 224 | 205 | 231 | 226 | 224 | 208 | 194 | 2,352 | -0,019 |
| Warmińsko-mazurskie | 172 | 166 | 177 | 197 | 229 | 188 | 153 | 187 | 2,236 | 0,023 |
| Wielkopolskie | 198 | 189 | 168 | 182 | 166 | 175 | 168 | 157 | 2,297 | -0,030 |
| Zachodniopomorskie | 196 | 187 | 182 | 195 | 202 | 205 | 198 | 183 | 2,292 | -0,009 |
