# Supplementary material for: Determinants and disparities in access to paediatricians in Poland
Source: BMC Prim Care. 2022 Apr 27;23:94. doi: 10.1186/s12875-022-01701-2 (PMC9044810; doi:10.1186/s12875-022-01701-2)
Supplement: Supplementary file 3 — Additional file 3. A – 3 Attributive variables (2017) [file 12875_2022_1701_MOESM3_ESM.docx]

A – 3 Attributive variables (2017)

| Voivodships | Population  0-17 years old | Population  density  0-17 years old | Increase  rate  of population  0-17 [%] | Income in PLN  in disposal  per capita | Number of hospital beds per 10000 residents | Paediatrician workforce per 100000 residents under 17 years of age | Treated on paediatric wards (including inter-ward movement) | Degree of urbanization | No of treatment per  paediatrician |
| --- | --- | --- | --- | --- | --- | --- | --- | --- | --- |
| Dolnośląskie | 490609 | 24,7 | -3,3 | 1626 | 50 | 54 | 31277 | 68,8 | 118 |
| Kujawsko-pomorskie | 376227 | 21,0 | -7,5 | 1457 | 47 | 62 | 25687 | 59,3 | 109 |
| Lubelskie | 376198 | 15,0 | -10,2 | 1437 | 53 | 38 | 19258 | 46,5 | 135 |
| Lubuskie | 183691 | 13,2 | -6,1 | 1591 | 43 | 47 | 10713 | 64,9 | 125 |
| Łódzkie | 417936 | 23,0 | -6,0 | 1566 | 52 | 80 | 23493 | 62,7 | 70 |
| Małopolskie | 642456 | 42,2 | -3,2 | 1492 | 44 | 52 | 36443 | 48,3 | 110 |
| Mazowieckie | 1017531 | 28,5 | 3,1 | 1912 | 48 | 56 | 64433 | 64,3 | 112 |
| Opolskie | 157829 | 16,7 | -9,4 | 1511 | 46 | 48 | 11376 | 52,8 | 150 |
| Podkarpackie | 389238 | 21,8 | -9,7 | 1254 | 48 | 48 | 29750 | 41,2 | 158 |
| Podlaskie | 206019 | 10,3 | -10,1 | 1586 | 50 | 42 | 19828 | 60,7 | 228 |
| Pomorskie | 454426 | 24,8 | -0,6 | 1649 | 40 | 54 | 27539 | 63,9 | 113 |
| Śląskie | 770744 | 62,5 | -3,6 | 1646 | 55 | 52 | 38616 | 76,9 | 96 |
| Świętokrzyskie | 207297 | 17,8 | -11,5 | 1433 | 49 | 50 | 20188 | 44,6 | 194 |
| Warmińsko-mazurskie | 263322 | 10,8 | -9,2 | 1496 | 47 | 46 | 22849 | 59,0 | 187 |
| Wielkopolskie | 672300 | 22,5 | -1,8 | 1607 | 45 | 36 | 38270 | 54,6 | 157 |
| Zachodniopomorskie | 294829 | 13,0 | -7,6 | 1653 | 46 | 50 | 27010 | 68,6 | 183 |
| Statistic parameters | | | | | | | | | |
| Mean | 432 541 | 23 | -6 | 1 557 | 48 | 51 | 27 921 | 59 | 140 |
| Median | 382 733 | 21 | -7 | 1 576 | 48 | 50 | 26 349 | 60 | 130 |
| Standard deviation | 231 499 | 13 | 4 | 138 | 4 | 10 | 12 408 | 10 | 41 |
| Minimum | 157 829 | 10 | -11 | 1 254 | 40 | 36 | 10 713 | 41 | 70 |
| Maximum | 1 017 531 | 63 | 3 | 1 912 | 55 | 80 | 64 433 | 77 | 228 |
