# Supplementary material for: Determinants and disparities in access to paediatricians in Poland
Source: BMC Prim Care. 2022 Apr 27;23:94. doi: 10.1186/s12875-022-01701-2 (PMC9044810; doi:10.1186/s12875-022-01701-2)
Supplement: Supplementary file 4 — Additional file 4. A – 4A The taxonomic accessibility model variables [file 12875_2022_1701_MOESM4_ESM.docx]

A – 4A The taxonomic accessibility model variables

| Voivodship | Population  0-17 years old | Paediatrician workforce per 100000 residents under 17 years of age | Treated on paediatric wards (including inter-ward movement) | Average income in disposal per capita | Out-patient  departments | No of emergency  rescue teams | Average monthly gross wages and salaries in human health and social work activities | Railway lines operated per 100 km2 | Hard surface public roads total per 100 km2 | Passenger cars  per 1000 population | National regular  transport lines in km | Expenditure of provinces for health care  in millions PLN |
| --- | --- | --- | --- | --- | --- | --- | --- | --- | --- | --- | --- | --- |
|  | x1 | x2 | x3 | x4 | x5 | x6 | x7 | x8 | x9 | x10 | x11 | x12 |
| Dolnośląskie | 490609 | 54 | 38270 | 1626 | 1542 | 116 | 4001 | 8,7 | 98,5 | 603 | 43889 | 49,7 |
| Kujawsko-pomorskie | 376227 | 62 | 11376 | 1457 | 862 | 88 | 3765 | 6,7 | 102,1 | 573 | 84311 | 43,9 |
| Lubelskie | 376198 | 38 | 19258 | 1437 | 1288 | 89 | 3941 | 4,2 | 88,7 | 567 | 56230 | 11,7 |
| Lubuskie | 183691 | 47 | 20188 | 1591 | 585 | 51 | 3814 | 6,3 | 63,3 | 619 | 25521 | 41,3 |
| Łódzkie | 417936 | 80 | 19828 | 1566 | 1621 | 98 | 4055 | 5,9 | 113,7 | 592 | 39596 | 18,3 |
| Małopolskie | 642456 | 52 | 10713 | 1492 | 1915 | 130 | 4024 | 7,1 | 170,6 | 554 | 7396 | 23,0 |
| Mazowieckie | 1017531 | 56 | 38616 | 1912 | 3028 | 193 | 4390 | 4,8 | 106,8 | 648 | 122768 | 239,6 |
| Opolskie | 157829 | 48 | 23493 | 1511 | 546 | 42 | 4029 | 8,2 | 90,3 | 626 | 12845 | 5,1 |
| Podkarpackie | 389238 | 48 | 31277 | 1254 | 1242 | 86 | 3729 | 5,0 | 94,2 | 532 | 38944 | 82,3 |
| Podlaskie | 206019 | 42 | 29750 | 1586 | 764 | 55 | 3760 | 3,6 | 66,2 | 503 | 14902 | 32,2 |
| Pomorskie | 454426 | 54 | 27539 | 1649 | 1026 | 89 | 4058 | 7,0 | 75,9 | 577 | 32647 | 85,7 |
| Śląskie | 770744 | 52 | 27010 | 1646 | 2849 | 160 | 3974 | 15,8 | 176,8 | 563 | 26004 | 65,0 |
| Świętokrzyskie | 207297 | 50 | 25687 | 1433 | 624 | 46 | 4035 | 6,2 | 123,4 | 546 | 14902 | 17,2 |
| Warmińsko-mazurskie | 263322 | 46 | 64433 | 1496 | 869 | 77 | 3723 | 4,5 | 56,4 | 524 | 31815 | 7,3 |
| Wielkopolskie | 672300 | 36 | 36443 | 1607 | 1931 | 117 | 3970 | 6,3 | 98,8 | 648 | 39492 | 119,9 |
| Zachodniopomorskie | 294829 | 50 | 22849 | 1653 | 973 | 82 | 3774 | 5,1 | 61,2 | 555 | 44105 | 21,6 |
| Statistic parameter and taxonomic model | | | | | | | | | | | | |
| Mean | 432540,8 | 51 | 27921 | 1557 | 1354 | 95 | 3940 | 6,6 | 99,2 | 577 | 41364 | 54,0 |
| Standard deviation | 231498,9 | 10 | 12408 | 138 | 736 | 40 | 169 | 2,7 | 33,9 | 42 | 28331 | 57,3 |
| Minimum | 157829 | 36 | 10713 | 1254 | 546 | 42 | 3723 | 3,6 | 56,4 | 503 | 7396 | 5,1 |
| Maximum | 1017531 | 80 | 64433 | 1912 | 3028 | 193 | 4390 | 15,8 | 176,8 | 648 | 122768 | 239,6 |
| Model | 1017531 | 80 | 64433 | 1912 | 3028 | 193 | 4390 | 15,8 | 176,8 | 648 | 122768 | 239,6 |
| Model u* | 2,3 | 2,7 | 2,1 | 2,1 | 2,0 | 1,9 | 2,3 | 2,6 | 2,3 | 1,1 | 2,8 | 3,3 |

A – 4B Standardized variables, distance to the model and taxonomic measure for each unit (paediatric service accessibility index-PSA)

| Voivodship | x1 | x2 | x3 | x4 | x5 | x6 | x7 | x8 | x9 | x10 | x11 | x12 | Distance | PSA |
| --- | --- | --- | --- | --- | --- | --- | --- | --- | --- | --- | --- | --- | --- | --- |
| Dolnośląskie | 0,251 | 0,300 | 0,834 | 0,502 | 0,255 | 0,528 | 0,359 | 0,771 | -0,020 | 0,627 | 0,089 | -0,075 | 7,094 | 0,742 |
| Kujawsko-pomorskie | -0,243 | 1,158 | -1,333 | -0,731 | -0,669 | -0,174 | -1,033 | 0,041 | 0,086 | -0,093 | 1,516 | -0,175 | 8,817 | 0,679 |
| Lubelskie | -0,243 | -1,325 | -0,698 | -0,876 | -0,090 | -0,149 | 0,001 | -0,872 | -0,309 | -0,237 | 0,525 | -0,738 | 9,747 | 0,645 |
| Lubuskie | -1,075 | -0,431 | -0,623 | 0,248 | -1,045 | -1,102 | -0,748 | -0,105 | -1,058 | 1,012 | -0,559 | -0,222 | 10,089 | 0,633 |
| Łódzkie | -0,063 | 2,956 | -0,652 | 0,065 | 0,363 | 0,077 | 0,680 | -0,251 | 0,428 | 0,363 | -0,062 | -0,624 | 7,862 | 0,714 |
| Małopolskie | 0,907 | 0,047 | -1,387 | -0,474 | 0,762 | 0,880 | 0,497 | 0,187 | 2,106 | -0,549 | -1,199 | -0,542 | 8,520 | 0,690 |
| Mazowieckie | 2,527 | 0,534 | 0,862 | 2,581 | 2,275 | 2,460 | 2,662 | -0,652 | 0,225 | 1,708 | 2,873 | 3,241 | 4,703 | 0,829 |
| Opolskie | -1,187 | -0,295 | -0,357 | -0,337 | -1,098 | -1,328 | 0,522 | 0,589 | -0,262 | 1,180 | -1,007 | -0,853 | 9,925 | 0,638 |
| Podkarpackie | -0,187 | -0,280 | 0,271 | -2,202 | -0,152 | -0,224 | -1,247 | -0,579 | -0,147 | -1,078 | -0,085 | 0,494 | 9,762 | 0,644 |
| Podlaskie | -0,979 | -0,897 | 0,147 | 0,207 | -0,802 | -1,002 | -1,066 | -1,091 | -0,973 | -1,774 | -1,460 | -0,381 | 11,074 | 0,597 |
| Pomorskie | 0,095 | 0,268 | -0,031 | 0,668 | -0,446 | -0,149 | 0,699 | 0,151 | -0,687 | 0,003 | -0,308 | 0,553 | 7,956 | 0,710 |
| Śląskie | 1,461 | 0,138 | -0,073 | 0,644 | 2,032 | 1,632 | 0,201 | 3,363 | 2,289 | -0,333 | -0,542 | 0,193 | 6,461 | 0,765 |
| Świętokrzyskie | -0,973 | -0,090 | -0,180 | -0,905 | -0,992 | -1,228 | 0,562 | -0,141 | 0,714 | -0,741 | -0,934 | -0,643 | 9,856 | 0,641 |
| Warmińsko-mazurskie | -0,731 | -0,480 | 2,943 | -0,443 | -0,659 | -0,450 | -1,284 | -0,762 | -1,262 | -1,270 | -0,337 | -0,815 | 10,391 | 0,622 |
| Wielkopolskie | 1,036 | -1,515 | 0,687 | 0,360 | 0,784 | 0,553 | 0,177 | -0,105 | -0,011 | 1,708 | -0,066 | 1,152 | 7,597 | 0,723 |
| Zachodniopomorskie | -0,595 | -0,087 | -0,409 | 0,694 | -0,518 | -0,325 | -0,981 | -0,543 | -1,120 | -0,525 | 0,097 | -0,565 | 9,632 | 0,649 |
